# Autologous stem cell transplantation for a monoclonal gammopathy of undetermined significance mimicking amyotrophic lateral sclerosis: A case report

**DOI:** 10.3892/etm.2014.1814

**Published:** 2014-06-27

**Authors:** LINNA XIE, FANG ZHOU

**Affiliations:** Department of Hematology, The General Hospital of Jinan Military District, Jinan, Shandong 250031, P.R. China

**Keywords:** auto stem cell transplant, monoclonal gammopathy of undetermined significance

## Abstract

It is rare for patients with monoclonal gammopathy of undetermined significance (MGUS) to present with clinical features of fatal motor neuron disease, for example amyotrophic lateral sclerosis (ALS). There is no standard and effective therapy for either MGUS or ALS. In addition, stem cell transplantation appears to be ineffective for the treatment of this disease. In the present study, a 47-year old female with MGUS that mimicked ALS is presented. The M-protein levels of the patient were normalized following two cycles of chemotherapy and autologous stem cell transplantation treatment. MGUS was found to be alleviated and the symptoms of ALS did not deteriorate. The results showed a positive therapeutic effect of autologous stem cell transplantation for MGUS.

## Introduction

Monoclonal gammopathy of unknown significance (MGUS) is an asymptomatic plasma cell dyscrasia, which is characterized by the presence of M-protein in individuals lacking evidence of multiple myeloma (1).

Numerous studies have found underlying pathological associations between neuropathies and MGUS. Peripheral neuropathies have been found in 8–36% of patients with MGUS (2) and in 50% of patients with immunoglobulin M (IgM)-MGUS (3). These patients showed a slowly progressive, sensory motor demyelinating neuropathy with antibodies and T-cell infiltration in the nerve lesions (4,5).

In the present study, a patient with IgG-MGUS, with motor neuron disorder as the main complaint, mimicking amyotrophic lateral sclerosis (ALS), is reported. The response of the patient to chemotherapy and autologous stem cell transplantation (ASCT) is presented.

## Case report

A 47-year-old Chinese female was admitted to the General Hospital of Jinan Military District (Jinan, China) due to a 10-month history of muscle weakness and progressive muscle wasting of the limbs and a 3-month history of slurred speech and involuntary muscle twitching. The therapy protocols described in this study were approved by Ethics Committee, Institute of the General Hospital of Jinnan Military. The patient’s legal guardian signed written informed consent before the therapy.

The neurological examination revealed atrophy in the left first dorsal interossei (DI) muscle and left thenar muscle. Fasciculation was observed in the left first DI, left biceps and lingual muscle. The results from the strength tests, presented as Medical Research Council grades (6) were as follows: 5-/5 for the left extensor carpi radialis longus and 4/5 for the left infraspinatus. Diminished grip strength of the left hand was observed (4/5); however, normal strength (5/5) was found for the right arm and bilateral lower extremities. The deep tendon reflexes were 3/4 in the left brachioradialis, biceps and triceps, and 2/4 for others. The Hoffmann and Babinski signs were negative. The sensory examination was intact and no coordination deficits or cranial nerve deficits were observed in the patient.

An electrodiagnostic study revealed low amplitudes in the compound muscle action potentials of the left upper extremity motor nerve, with intact sensory nerve action potential responses. There was no evidence of any abnormal temporal dispersion or conduction block in multiple nerves tested. There were 1–2+ fibrillation potentials and positive sharp waves in the left tibialis anterior and first dorsal interosseous muscles. Magnetic resonance imaging examination revealed Wallerian degeneration of the bilateral thalamus, the midbrain, the lateral cerebral ventricle and the centrum ovale. The patient showed clinically possible amyotrophic lateral sclerosis (ALS). However, an exact diagnosis that would account for the clinical condition was not identified.

Further laboratory investigations revealed elevated IgG 2,500 mg/dl (normal, 751–1,560 mg/dl) and κ light chain 2,990 mg/dl (normal, 629–1,350 mg/dl) levels in the serum. The level of IgG in the cerebrospinal fluid (CSF) was also elevated 52.8 mg/l (normal, 0–34 mg/l) and CSF-restricted IgG oligoclonal bands were positive. Immunofixation electrophoresis of serum and CSF showed the same positive IgG protein. The myelin-basic-protein antibody (MBP.Ab) level was 1.203 μg/l in the serum (normal, <0.650 μg/l) and 1.411 μg/l in the CSF (normal, <0.750 μg/l); and the myelin-oligodendrocyte-glycoprotein antibody (MOG.Ab) level was 1.484 μg/l in the serum (normal, <0.560 μg/l) and 1.424 μg/l in the CSF (normal, <0.640 μg/l). The increased levels of antibodies indicated an association between neuropathy and IgG-MGUS. Bence-Jones protein was not detected and proteinuria was 2.5 g/day. B12 deficiency and hyperparathyroidism were excluded. The results from ^99m^Tc methyl diphosphonate (MDP) bone scintigraphy and fludeoxyglucose positron emission tomography-computed tomography demonstrated that there was no positive manifestation. Bone marrow (BM) examination revealed 5% atypical plasma cells, which were found to express surface cluster of differentiation (CD)38, CD34, CD117, human leukocyte antigen (HLA)-DR, CD33, CD19 and CD13. The chromosomal constitution was 46, XX. Fluorescence *in situ* hybridization analyses of p53, RB1, 1q21, D135319 and IGH gene rearrangements were negative.

**Figure 1:**
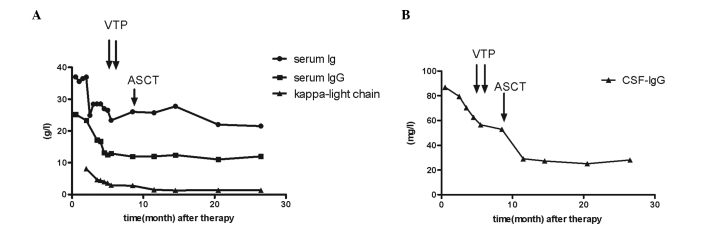
Time line of clinical response to the therapeutic treatments. (A) Serum IgG levels decreased following chemotherapy and ASCT; the IgG and κ-light chain levels were decreased rapidly during the therapy course. (B) The level of CSF-IgG markedly declined following ASCT, and remained low. IgG, immunoglobulin G; ASCT, autologous stem cell transplantation.

The patient showed no signs of end-organ damage, the serum M-protein level of the patient was 2.5 g/dl (normal, <3 g/dl) and the clonal plasma cell population in the BM was 5% (normal, <10%); in combination, these results support the diagnosis of MGUS. Chemotherapy with vascular-targeted photodynamic therapy (VTP) and bortezomib, plus dexamethasone and thalidomide, was administered due to the worsened motor neuron damage caused by M-protein. Following two cycles of chemotherapy, the serum IgG levels were reduced and the percentage of atypical cells decreased to 0.7%. Dexamethasone (5 mg) and cytarabine (50 mg) treatment was then administered by intrathecal injection. The levels of IgG in the CSF were found to decrease significantly. The patient was administered cyclophosphamide (2 g/m^2^, 2 days) as mobilization chemotherapy, followed by ASCT; the conditioning treatment consisted of 900 cGy total body irradiation (TBI) and melphalan 140 mg/m^2^. Three months following ASCT, the atrophy in the left first DI and left thenar muscles was persistent. The results from the strength tests were 4-/5 for the biceps and 4-/5 for the triceps. The wrist extensors on the left side were weakened with an inability to extend the fingers. In addition, fasciculation in the upper extremity muscles was observed with a scattered distribution, predominantly proximally. Serum IgG and κ light chain test results remained positive (Fig 1). Thus, oral treatment of the patient with thalidomide was resumed. During the 24-month follow-up, the patient’s ALS mimicking symptoms did not appear to be aggravated, and the results from the strength tests were 4-/5- for the biceps and 4-/5- for the triceps. Cranial nerve deficits were observed, and the patient exhibited mild dysarthria and dysphasia.

## Discussion

The prudent management strategy of MGUS is ‘watch and wait’. At present, there are no standard treatments. Patients with MGUS with clinical features of ALS are rare. In the present case, serum-IgG and CSF-IgG levels were elevated, and immunofixation electrophoresis of immunoglobulin showed that serum and CSF IgG were homologous. This result indicates that the IgG in the CSF may not be endogenous; it may originate from M-protein in the serum that passed through the damaged blood-brain barrier (BBB). The elevated levels of MBP.Ab and MOG.Ab are also indicative of damage of the CNS.

The serum levels of IgG and the plasma cell levels were reduced following chemotherapy; however, the IgG levels in the CSF remained high. This may be due to the chemotherapy administered not being able to penetrate the BBB, or slow attenuation of IgG in the CSF Following the intrathecal injection of dexamethasone and cytarabine, the IgG levels in the CSF decreased significantly. The symptoms of lingual muscle fasciculation and slurred speech were ameliorated. TBI was used in the conditioning regimen, which may have helped to eliminate autoimmune damage of the motor neurons caused by M-protein. However, there were no significant improvements in neuronal symptoms following ASCT. The patient did not benefit from single ASCT. This may be as a result of the malignant clones not being completely cleansed, or the nervous system damage being irreversible. Patients with multiple myeloma who achieve complete remission (CR) with conventional chemotherapy and do not receive transplantation have the same prolonged progression-free survival and overall survival as those attaining CR following ASCT (7). Conservative chemotherapy or double ASCT may be used for the patient as a follow-up treatment.

MGUS mimicking ALS is extremely rare. The ALS symptoms of the patient in the present study aggravated quickly. Although the therapies did not achieve the expected effect, they delayed the deterioration of the disease. These observations may be of use for the treatment of MGUS in the future.
